# Exploring adolescents’ experiences navigating the intersection of their gender, sport, and dietary identities: an interpretative phenomenological study

**DOI:** 10.3389/fnut.2024.1524135

**Published:** 2024-12-19

**Authors:** Alysha L. Deslippe, Georgia Middleton, Olivia Y. Wu, Coralie Bergeron, Tamara R. Cohen

**Affiliations:** ^1^Human Nutrition, Faculty of Land and Food Systems, University of British Columbia, Vancouver, BC, Canada; ^2^Healthy Starts, British Columbia Children’s Hospital Research Institute, Vancouver, BC, Canada; ^3^College of Nursing and Health Sciences, Flinders University, Adelaide, SA, Australia; ^4^Women and Children’s Health Sciences, Faculty of Medicine, University of British Columbia, Vancouver, BC, Canada; ^5^Food, Nutrition and Health, Faculty of Land and Food Systems, University of British Columbia, Vancouver, BC, Canada

**Keywords:** dietary behaviors, adolescents, gender, motivation, athletes

## Abstract

Not all adolescents have positive sport experiences. Research has repeatedly identified ties between unfavorable eating patterns and food beliefs (i.e., a dietary identity) that hinder an athletes’ health and performance. Gender norms and pressures over idealized bodies (e.g., boys are muscular whereas girls are thin) play a critical role in the manifestation of unfavorable eating habits. However, most research has focused on the experiences of athlete girls in elite sport spaces (e.g., high performance), leaving gaps in our understanding of how diverse youth in high school spaces are impacted. To address this gap, we aimed to explore the intersections between adolescents’ sport, dietary and gender identities in high school. We conducted 33 interviews with high school athletes (*n* = 9 girls and *n* = 9 boys) and non-athletes (*n* = 4 non-binary, *n* = 6 boys and *n* = 5 girls) using methods informed by an interpretative phenomenological approach. We generated three themes capturing adolescents’ experiences: (1) *De-gendering protein and muscles;* (2) *Food displays what I value;* and (3) *Being [too] masculine is bad.* Athletes recognized ties between masculinity and sport, but spoke about traditionally masculine eating habits (e.g., valuing protein) and body ideals (e.g., being muscular) as part of their athletic identity, not gender. Regardless of sport involvement, adolescents altered their outward expression of their dietary habits to demonstrate pieces of their identities, like being an athlete (e.g., eating protein) or feminine (e.g., smaller bites). Adolescents also spoke about using food to pursue idealized bodies that show deviation from traditional gender norms. Finally, adolescents held conflicting views about masculinity as being both toxic and useful for competition. By understanding identity intersections, coaches, trainers, parents, and sport decisions-makers can make more informed decisions about sport policy, programs, and practice that involve dietary advice to support this population.

## 1 Introduction

Sports have positive impacts on an adolescents (13–18 years) development (e.g., physical and mental health) (1). However not all sport experiences are positive. Athletes experience pressures over what and how much they eat with the guise of improving performance (2, 3). In many cases these pressures result in the adoption of a dietary identity (e.g., food habits and beliefs) that does not meet elevated nutrient needs for sport (e.g., calories), hindering performance and increasing injury risk (4, 5).

An individual’s ‘personal food system’ can dictate their dietary behaviors and is informed by their physical environment, social and cultural norms, biology and psychological factors (6). Importantly, desires to portray a certain dietary identity, such as the identity of an athlete, can influence the uptake of long lasting dietary patterns (6). For example, in a cross-sectional survey of 348 teen athletes from four countries competing at the international level, 82.2% of teens used a sport supplement (7). Despite this, most athletes reported low knowledge on what the sport supplements they used were for and were motivated by improving performance based on messages they had heard from coaches, peers or media sources (7). Overreliance on supplements instead of whole foods among athletes is problematic as it can lead to lower overall energy intake and lack of consumption of other critical nutrients that are part of a foods matrix (8), demonstrating the complex interaction of eating for sports performance compared to health. Further, more athlete boys used protein supplements and were motivated to alter what they ate to gain muscle whereas more athlete girls used vitamins or minerals under the motivation of improving general health (7). Such pattern points toward the notion of young athletes following beliefs about what an athlete ‘should’ do and these beliefs are often rooted in gender norms (7, 9, 10). It is important to understand how athletic, dietary and gender identities among athletes intersect to reveal pivotal moments where intervention can be used to improve sport experiences for all athletes through food (6). Historically, research has focused on higher rates of poorer sport experiences and its interplay with food relationships among athlete girls, making it imperative to clarify the unique experiences of boys or gender diverse youth (11, 12). It is especially salient to explore this in the high school context as these athletes rarely have access to trained coaches or dietitians who can help guide eating (7, 10).

Sport involvement and gender identities have been historically linked with belief that sport is masculine (13, 14). Since the 1930s, these beliefs have been challenged by social movements for gender equality (13). This shift in social norms around sport has contributed to greater opportunities for women and girls, (13) but, sport experiences remain unequal. Women in sports are paid less (15, 16) and report worse mental health outcomes compared to men in sports (17, 18). Inequities in sport experiences have been observed in settings as early as adolescence, with girls more likely than boys to drop out from sport (19, 20) and non-binary youth less likely to participate at all (21). While current initiatives focus on improving equality in sport by creating more opportunities for participation, little research has focused on fostering equity in sport experiences. This is problematic as ‘separatism’ of sport based on sex in it of itself is not enough to challenge or disrupt gender norms that transcend across sports (14). Such an approach conflates sexes (biological) and gender (societal) pressures diverse athletes experience as one, assuming that they can both be resolved by creating specific spaces for male and female athletes. By failing to account for the overwhelming pressures an athlete may face due to their gender identity regardless of their sex (14), sport experiences may remain unequal. Thus, research exploring how challenging social norms impacts sport experiences, (13) including intersections between sport, gender and dietary identities, is needed.

The intersectionality between an individuals’ athletic, dietary, and gender identities impact their sport participation, (19, 21) dietary intake, (3, 22) and consequently sport experiences (23). For example, athlete boys are more likely than athlete girls to consume protein and protein supplements with the intent to gain muscle, (7, 24) attributes traditionally associated with masculine identities (12, 25). In contrast, athlete girls report greater tendencies to restrict food intake to appear feminine and avoid gaining bulk while competing (3, 4, 12, 22). Outside of sport participation, gender norms surrounding dietary identities have also been reported with feminine individuals more likely to avoid meat, consume smaller portions and choose ‘healthy’ low calorie foods like produce (26). Intersections between dietary and gender identities can disadvantage youth in gendered ways, contributing to unique nutritional risks and likelihood of sport drop-out (12, 22, 25).

Recent work by Schaillée et al. (27) reports that while adolescents understand gender disparity in sport, they do not always oppose it. This demonstrates the complexity of intersectionality between gender, dietary and sport identities. Building on the belief that access to sport participation is not enough to make sport equitable, (13) we aim to explore what gender means in the context of sports, through intersections between adolescents’ athletic, dietary, and gender identities using an interpretative phenomenological approach. As no literature has explored these intersections, this study will address a key gap needed to inform our understanding of what more equitable sport experiences may require when it comes to dietary supports.

## 2 Methods

### 2.1 Study design

As sport, gender, and dietary habits are unique human experiences shaped by cultural and social norms, we followed an interpretive paradigm, informed by bounded relativism ontology (i.e., multiple realities exist and are rooted in personal context), and an epistemological orientation of social constructionism (i.e., reality is socially constructed) (28). To effectively explore the interplay between athletic, dietary, and gender identities, this study took an Interpretive Phenomenological Approach (IPA) (29). IPA facilitates exploration of an ‘insider look’ into adolescents’ experiences (30). As such, we focused on a first-person perspective to make sense of lived structures and experiences, uncovering the ‘essences’ of how adolescents decide what to eat at the intersection of their gender, athletic, and dietary identities (31, 32). As IPA has been used to explore gender, sex, eating disorders, and sport psychology, it is well-suited for our research aim (33). This IPA study is part of a bigger study the EATing in a GENdered world study (EatGen), a mixed-methods project focused on co-designing inclusive interventions to support athletes’ eating habits in schools. Ethical approval for the project was provided by the University of British Columbia Research Ethics board (H22-00121). This paper focuses exclusively on the qualitative interview results pertaining to experiences navigating intersections between athletic, dietary, and gender identities following best-practice qualitative methods (Supplementary Table 1).

### 2.2 Recruitment

Adolescents who attended a secondary school in British Columbia, Canada (grades 8–12) were recruited. The school is situated in a suburban community with predominately high income, two-parent households (34). This school was chosen due to its diverse athletic program and existing relationship with the research team.

Adolescents were recruited by posters and in-person presentations by two female research assistants who had no former relationships with the school or adolescents. At two time points (April–June 2022 and December 2022 – January 2023), interested adolescents scanned a QR code to provide their contact information to the research team. Eligible participants provided informed consent over the phone. Parents of adolescents under 18 years of age were also called to provide consent for their children. Adolescents were excluded if they were: pregnant, living with an eating disorder, on exchange, diagnosed with a severe dietary restriction (e.g., Crohns), and/or did not have access to technology to participate in a virtual interview. We purposively recruited adolescents to balance sex (male/female), as sports compete based on sex, and sport involvement (athlete/non-athlete) (35). To be considered an athlete, an adolescent had to have participated in a school sport and/or club sport (i.e., practices held after school with competition against other local schools/club teams) in the past year. Differing from traditional phenomenology work, we recruited a larger sample of adolescents (29) to provide insight into differences between groups and meet saturation for our broader projects objectives that interested teens were made aware of.

### 2.3 Interview guide

Aligned with an interpretive phenomenological approach, an open-ended interview guide (35) was developed by the first and last author based on concepts from behaviour change theory (36) and the Food Literacy Competencies for Young Adults (37) framework. After pilot-testing the guide with one adolescent, two questions were altered to ensure language was appropriate for the target population (35). This study focused on responses to questions about beliefs and experiences relating to dietary habits, gender, and sports. See Supplementary Table 2 for the interview guide used in the broader project.

Adolescents were asked to define their view of masculinity and femininity in their own words at the start of the interview. After this, adolescents self-identified how masculine or feminine they perceived themselves to be using a scale of 1 (masculine) to 10 (feminine). We elected to use this novel approach as more traditional measures, like the Bem Sex-Role inventory, are lengthy and restrictive (38) and do not facilitate our goal of understanding personal experience of identities. Thus, we aimed to facilitate a truer experience of what being masculine or feminine meant to each individual teen by using an open ended, self-identified approach to gender identity instead of using pre-determined definitions or categories.

### 2.4 Data collection

Demographic information was collected through an online survey. Adolescents then participated in a semi-structured virtual interview in a private location lasting ~30 min (19–57 min). Audio recordings of the interviews were transcribed verbatim by a transcription service, and de-identified. The interviewers wrote case notes following each interview. Adolescents received a $30.00 CAD gift card for participating.

All three interviewers were trained in how to probe for adolescents’ experiences by the first author, a certified facilitator. After two interviews each, the first author listened to the audio and provided feedback on interviewers’ techniques to improve the consistency and depth of responses across interviewers. Interviewers were not previously known to participants and were all female university level students who had never competed in competitive sports.

Three participants were excluded from the final sample, and their interviews excluded from analysis, as they no longer met eligibility criteria after implying they had previously lived with an eating disorder during their interview.

### 2.5 Analysis

Aligned with IPA, analysis started with open coding of transcripts line by line, where each line of data was assessed for ‘codes’ to indicating meaning in adolescents’ experiences. All coding was done inductively (i.e., from the data itself) to allow for true representation of participants experiences to guide the process (29, 39). Similar codes were then grouped together and considered for overarching commonalities within a transcript. Next, tentative overarching themes where formed (39). This process was repeated on the next transcript and so on in an idiographic nature (29). Once all transcripts were coded, differences in experiences were compared to confirm themes across adolescents experiences (39). The first author, a female researcher who previously competed in competitive sport and is now a coach of competitive high school athletes, led the analysis. The process of ‘bracketing’, where one intentionally tries to set aside their own beliefs and assumptions, (29, 39) was done in two ways. First, the lead author, who did not interact with participants and therefore had a degree of separation, led the analysis and discussed developing codes and themes with an individual of a different sex who had previous competed in sports and was not involved in the study. Second, an additional coder who did not play competitive sports reviewed all transcripts (authors two or three) during the open coding and theme consolidation stages.

## 3 Results

### 3.1 Participants

Thirty-three adolescents participated (Table 1). Of these, 18 were athletes (*n* = 9 girls; *n* = 9 boys) and 15 were non-athletes (*n* = 4 non-binary; *n* = 5 girls; *n* = 6 boys). Self-identity ratings (1 = masculine and 10 = feminine) in each group were: 1.5 among athlete boys, 7.5 among non-athlete boys, 6.5 among athlete girls and 8.0 among non-athlete girls. No non-binary non-athletes gave the same self-identity rating. Eleven athletes played a club and a school sport and seven played only a club sport.

**Table 1:** Characteristics of interviewed adolescents (*n* = 33).

ID	Gender	Self-identity	Ethnicity	Grade	Sports played
Athletes					
01	Girl	6.5	White	11	Basketball, cross country
02	Girl	9.5	White	10	Softball, volleyball
03	Boy	1.5	White	10	Baseball, hockey, volleyball
04	Boy	3.5	Mixed	9	Baseball
07	Girl	6.5	White	10	Aquatics, gymnastics, skating
08	Girl	6.5	White	12	Rowing
15	Boy	2.5	Mixed	10	Basketball, football
17	Girl	6.5	Chinese	10	Aquatics, horseback riding
18	Boy	5	White	10	Soccer
20	Girl	5	White	10	Basketball, field hockey,
27	Boy	1.5	White	9	Basketball, football, soccer
31	Girl	8	White	9	Basketball, dance, volleyball
32	Boy	3.5	White	12	Basketball, rugby, volleyball
34	Boy	6	White	10	Hockey, soccer
34	Girl	6.5	White	8	Dance
36	Boy	1.5	White	11	Lacrosse
38	Boy	5	White	9	Baseball, soccer
39	Girl	6.5	Undisclosed	8	Aquatics, track and field, volleyball
Non-athletes					
05	Boy	7.5	White	12	None
06	Boy	8.5	White	9	None
10	Non-binary	3	White	11	None
12	Girl	5	White	11	None
16	Non-binary	7	White	12	None
19	Boy	7.5	Korean	10	None
26	Girl	8	White	10	None
30	Girl	8	White	10	None
31	Girl	6	Chinese	12	None
33	Boy	4.5	White	9	None
33	Non-binary	7.5	Mixed	9	None
35	Non-binary	6	White	9	None
36	Girl	9.5	Hispanic	10	None
37	Boy	5.5	White	12	None
39	Boy	3.5	Filipino	8	None

Self-identity was assigned based on adolescents’ self-identity of their perceived masculinity (1) or femininity (10). Terms were defined in their own words first and not outlined by the research team.

### 3.2 Themes

The IPA analysis generated three themes: (1) *De-gendering of protein and muscles;* (2) *Food displays what I value*; and (3) *Being [too] masculine is bad*. Differences in experiences between athletes and non-athletes from diverse genders are highlighted below. A summary of all themes and sub-themes can be found in Table 2 with additional quotes presented.

**Table 2:** Themes and sub-coding structure.

Theme	Sub-theme	Example
De-gendering of protein and muscles	Sports are manly	I think of like a man when I think of masculine energy like tough and sports. (Girl non-athlete #26, self-identity rating of 8.0)
	Fixation on protein	Before a sports thing, obviously you are supposed to eat… You got to get protein, protein bars, protein shakes. I make sure to get protein. (boy athlete #15, self-identity rating 2.5)
	Muscles are important	[in rugby] You are put somewhere dependent on your weight. If you are a bigger person, you are probably going to be a front. That’s just how it works… I always wanted bigger legs, but my legs just would not grow no matter how hard I worked them. So, I was a little bit self-conscious in them [rugby uniform]. (Boy athlete #32, self-identity rating of 3.5)
Food displays what I value	Feminine teens mind how they eat	I’ll eat maybe less, just because it’s more feminine to take like smaller bites. (Girl athlete #7, self-identity rating of 6.5)
	Weight is bad	I know that if eat like a lot of carbs I might gain weight fast and for the women it’s harder to lose that weight. (Girl non-athlete #36, self-identity rating of 9.5)
Being [too] masculine is bad	Masculinity is negative	Whenever I hear masculine, that just makes me think of what’s stereotypically seen as masculine. So, I do not even really look at it in a very good sense, because it immediately makes my brain go to thoughts of toxic masculinity. (Non-binary non-athlete #16, self-identity rating of 7.0)
	Masculinity has positives	[Masculinity is] I would say mainly stronger, bigger, stuff like that. Mainly strength I would say. (Boy athlete #34, self-identity rating of 6.0)
	It has to match my identity	For me sometimes I feel – I wake up and I feel masculine and I just wanna dress masculine, act masculine. (Girl non-athlete #30, self-identity rating of 8.0)

#### 3.2.1 De-gendering of protein and muscles

When discussing their eating habits, athletes of all genders placed substantially more value on protein-rich foods than non-athletes, a nutrient traditionally tied to masculinity.

I should be eating food that gives me energy so that I play good. Like high protein. If I’m going to the gym, I’ll have a protein shake. For games, I’ll eat more protein and stuff that will give me energy because I know I’m going to be working harder. (Girl athlete #2, self-identity rating 9.5).

I’m not totally sure if everything that I eat does have protein in it. I do believe that vegetables and such are the ones that have protein in them, correct? Not meat, right? (Boy non-athlete #33, self-identity rating of 4.5).

These contrasting reflections show the value that athletes place on protein, regardless of gender, for sports performance. This could indicate a shift away from a focus on protein as being masculine and instead part of athletic identity. This argument is supported by non-athletes’ discussion of protein as an important part of a balanced diet, but not their focus, and further, larger uncertain in this group about the nutritional role of protein and food sources of protein. This lack in knowledge diverges from athletes who had ample knowledge about the role of protein in sports performance and the foods that contained protein.

While the above results suggest a de-gendering of protein, some athlete girls continued to have a complex relationship with this nutrient.

I feel like steak is very masculine with food. I do not eat it. I was vegetarian for a while and I felt really drained all the time, like I wasn’t getting enough protein and stuff. Because a lot of protein powders have animal by-products in them. [Now] I do not eat red meat, but I do have protein in other ways, like eggs. (Girl athlete #7, self-identity rating 6.5).

This athlete girl recognizes the importance of protein, like red meat, in improving her athletic performance. However, she is also conflicted by the contrast between her feminine identity and her recognition of traditional norms surrounding red meat as masculine. The balance between not wanting to eat meat, yet not feeling like she can perform if she adopts a vegetarian identity and avoids meat, resulted in a change to her dietary identity to someone who consume other meats and animal products like eggs to balance her gender and athletic identity.

All athletes talked about muscular bodies as an important part of showing others their athletic identity, instead of an exclusively important trait for athlete boys. In contrast, no non-athletes talked about idealizing muscular bodies and needing to eat a certain way to pursue being muscular.

I lost my definition. So, I had to do ab work to get that back. I was trying to build muscle, so I cut down on sugar, ate more protein, stayed well hydrated. (Girl athlete #1, self-identity rating of 6.5).

I wanna get bigger for football and I like going to the gym so I wanna eat more food… I’m trying to gain weight. So, more food, right? (Boy athlete #15, self-identity rating of 2.5).

Athletes in this sample highlight the value of protein and pursuit of muscular bodies through their dietary identity as an athlete. These beliefs and actions arose regardless of gender identity, reinforcing the idea that athletic identities are tied to protein and muscular bodies, not gender identities.

#### 3.2.2 Food displays what I value

Adolescents who self-identified as feminine talked about changing eating habits in front of others to portray a traditionally feminine identity concerned with health and social image, regardless of sport involvement.

Gender has played a role on what I’m eating in front of [guys]. I’m not friends with very many guys, but I’m a lot more cautious about what I am eating - Quantity and usually what’s in the food that I’m eating, if it’s unhealthy or if it’s not balanced, I really think about that. (Non-athlete Boy #6, self-identity rating of 8.5).

Feminine pressures resulted in altered eating habits as a part of dietary identities to portray historically feminine behaviors like eating smaller portions and choosing healthier foods. These pressures transcended sex and sport involvement and were associated with personal views of femininity, an aspect of a teens gender identity.

Dietary identities (i.e., habits and motivations behind them) were also used to pursue traditionally idealized body shapes, like being thin. However, contrary to historical norms, all groups except athlete boys discussed pressures to control and avoid weight by altering what they ate.

My mom just made an offhand comment “do you really need that much food?” And after they had left the kitchen, I had started crying. I think the net change on myself has been positive because I lost weight... I do not remember how drastic I reduced what I was eating but it was a noticeable decrease. (Boy non-athlete #5, self-identity rating of 7.5).

Narratives just as this challenge traditional assumptions that social pressures to achieve smaller bodies primarily affect girls. Instead, adolescents of all genders who do not play sports navigated these pressures. Further, regardless of sport or sex, adolescents who identify as more feminine experience more felt pressure to alter their dietary in front of others to portray an outward feminine identity through what they eat.

#### 3.2.3 Being [too] masculine is bad

Adolescents expressed conflicted perspectives on masculinity, highlighting negative and positive attributes associated with masculine identities. For instance, this athlete girl hesitated when defining masculinity and prefaced her response by acknowledging the negative connotations surrounding masculine identities that may be associated by statement. The tension participants felt defining masculinity for fear of saying the wrong thing or perpetrating stereotypes was overt.

I do not know, it sounds so bad now that I say it. Like something bigger or tougher or less like soft and cozy [what masculine means]. (Girl athlete #8, self-identity rating of 6.5).

Other adolescents challenged stereotypical definitions of masculinity.

In society a lot of it tends to be toxic masculinity, not showing feelings and playing sports and all that. But a lot of my closest male friends, as well as my boyfriend, are very in touch with their feelings and are softer than I am. (Girl non-athlete #31, self-identity rating of 6).

These observations suggest that adolescents perceive a need for social norms surrounding masculinity to change beyond reductionist associations with physical strength, sports, and stoicism. However, not all adolescents shared this perspective. Some adolescents self-identifying as masculine, especially those who are also athletes, felt pride in their masculinity.

I think it, it’s pride for me. That’s what masculinity means. Because I try not to be a super masculine person because I know a lot of people get turned off from it in a social way. Dudes being super masculine and aggressive and all that.... I know there’s so many bad ways to use it, but I think you can look at it in a good way. (Boy athlete #32, self-identity rating of 3.5).

Yet even athletes such as this who identify as masculine and feel a sense of pride in that identity, still feel societal pressures to acknowledge the negative social connotations tied between masculinity and aggression. Dialog like this may signify a novel pressure that masculine athletes now place on themselves: avoid appearing too masculine and matching negative societal views despite personal pride felt in being masculine and posing some historically masculine traits. Moreover, negative views of masculinity and sports may deter other adolescents who do not identify as masculine from pursuing sport participation.

I know I do not fit at the [typical gender] spectrum [laughs], but you know, someone that maybe plays sports, goes to the gym, just has a very masculine ego… I’m not really masculine. (Non-athlete Boy #6, self-identity rating of 8.5).

Adolescents seem to be reluctant to participate in sport when they do not perceive their gender identity to match the historical masculine connotations associated with sport involvement. Additionally, adolescents who do identify with more masculine gender identities seem to experience this same hesitation in accepting a masculine identity with all its societal connotations as their own.

## 4 Discussion

This paper describes a novel exploration of the intersections between athletic, dietary, and gender identities from the perspective of adolescent athletes and non-athletes. Our findings revealed that masculine identities remain tied to sport involvement, but they are no longer linked exclusively to protein intake or muscularity as previously shown (26, 40). Instead, athletes perceived protein consumption and muscularity as a part of their athletic identities, and altered what they ate and how they viewed food as a result (i.e., their dietary identity). Further, our findings revealed shifts in how pursuit of idealized bodies and dietary identities are no longer represented as a ‘feminine problem’ (41); all adolescents who did not play sports, regardless of their gender (i.e., girls, boys and non-binary youth) or self-identity (i.e., masculine and feminine ratings) experienced pressures to avoid weight. Finally, our findings outline a complex picture of masculinity, as adolescents perceived masculinity as a source of pride and a deterrent for sport participation.

### 4.1 Is sport masculine?

In our sample, athletes expressed being more fixated on protein intake and achieving muscular body shapes compared to non-athletes. As literature suggests that gaining muscle and protein are traditionally tied with masculine identities, (7, 24, 25) our findings paint a new picture. Athletes in this sample view muscles and protein as an inherent part of being an athlete and not an inherent part of being masculine. Though promising in terms of challenging gender norms around dietary identities, some athlete girls in this sample still felt inner conflicted for consuming protein sources like red meat that carry traditional ties to masculinity (26, 40). Research conducted in collegiate athletes suggests that women balanced their muscular athletic build with feminine norms by choosing larger, more-masculine-appearing romantic partners (42). Taken in the context of our own findings, it is possible that athlete girls and women still experience underlying gender-based pressures that create tension between their own gender identities and athletic identities that manifest through dietary identities and idealized body shapes. However, the pressure athletes’ boys may experience in the context of their bodies should not be overlooked.

Our sample considered of a wide array of sports that are often associated with traditional masculinity like basketball and soccer (16). Recent literature exploring associations between dietary habits, mental health and injury risk in young adult dancers did not find significant differences in athletes’ body acceptance or dietary habits (43). However, female athletes did report higher self-esteem (43). This may suggest that male athletes experience greater stigmatization of their bodies compared to male athletes in a traditionally male sport like hockey (16), playing an unfavorable role in a male athletes’ perceptions of their bodies. Previous research supports this notion with adolescent male tootball players reported less body dissatisfaction compared to male runners (44). As we did not have the sample size to explore themes across individual sports, future research should explore how the intersection between sport, dietary and gender identities within different sports may vary to ensure the unique pressures boys (and girls) experience in sport are not overlooked.

### 4.2 Femininity is still linked to dietary identity

Adolescents highlighted social pressures related to femininity, but not masculinity, to directly impacting their dietary identities. This matches other literature where taking smaller bites, eating smaller portions and actively choosing ‘healthy’ foods are associated with a more feminine dietary identity (26). However, our study is one of the first to show that these pressure to eat ‘femininely’ are tied to personal identity (i.e., self-perception of how masculine or feminine an individual feels) and not gender identity (i.e., boy, girl or non-binary youth) or sex (i.e., male or female). This finding is important as current measurement of sex and gender in research exploring athletes’ experiences with food may not be sufficient to capture underlying norms. It is especially important as the push for more equal and equitable opportunities in sports sees greater inclusion of athletes of diverse sexes and genders training together (45). Team culture and social norms are key influencers in athletes’ food choices, (46) making it imperative that robust strategies to capture societal norms in dietary identities of athletes are developed. This will better ensure that athletes who need the most support in adopting a dietary identity focused on their performance needs instead of social norms can be identified.

### 4.3 Body shape is not just a feminine issue

Our findings on the intersection between dietary identities and gender identities through idealized bodies differ from previous literature, which suggests that weight is primarily a feminine issue (41). In this study, all non-athletes, including masculine self-identifying boys and non-binary adolescents, experienced pressure to avoid weight gain or lose weight. Further, athlete boys and girls experienced pressure to be muscular. Athletes’ focus on being muscular regardless of gender or self-identity likely comes from their lived experiences having their bodies constantly evaluated for performance (10). As such, embodied expectations to adopt a dietary identity consistent with promoting muscularity as part of athletic identity may place high school athletes at risk for disordered eating compared to non-athletes. Such observations have been corroborated in elite sport settings, (47) but not in high school sport settings until now.

### 4.4 Inclusion but not exclusion for all adolescents in sports

Many adolescents in our study described masculinity associated with negative personality and behavioral traits. In some cases, it resulted in adolescents in this sample feeling less inclined to participate in sports. This contrasts views from other adolescents who were mostly athlete boys in this study who identified masculinity as an entrenched, and positive part of their personal identities. Previous literature suggests that sport creates environments where historically masculine traits, like being competitive or aggressive, are viewed more favorably among boys (13) but not among other gender groups (42). Recent literature suggests that gender beliefs in sport are still present across a host of sports among collegiate athletes, with most sports associated as masculine or neutral, but not feminine (48). Further, the authors suggest that ‘gender typing sports’ (i.e., how gendered a sport is) may prevent athletes from participating or staying involved due to misalignment between their own gender identity and that of a particular sport, (48) a notion that may explain our findings here. As such, greater research is needed to understand how views of masculinity may hinder or support involvement in sports and for whom.

### 4.5 Practice implications

Our findings highlight the notion that de-gendering dietary and sport identities is important, but that there are caveats to be aware of. First, removing gender pressures around protein and muscularity as masculine are important, but have created a situation where all athletes, regardless of gender in our sample, fixated on protein as a part of athletic identity. This manifested as a use of proteins supplements (e.g., powders) instead of wholefoods. As the current position from sport experts and dietitians is to promote a ‘food first but not food only’ approach, athletes’ focus on protein supplements could be problematic for overall health (8). Second, the de-gendering of protein and muscles as a part of athletic identity might increase the risk of overlooking disordering eating among female athletes. For example, over-exercising under the label of athletic identity and increasing muscle might not be obviously interpreted by coaches, trainers or parents as a form of disorder eating. Coaches, trainers and clinicians in sport spaces need to be aware of gender sensitive tools to assess athletes’ eating habits in the context of sport such as the Athletes’ Relationship with Training scale (ART) (49). Finally, programs where athletes and non-athletes learn about food together (e.g., home economic classes) need to account for the impact of athletic identities to avoid inadvertently harming an athlete’s dietary identity. For example, prioritizing food as fuel to keep esthetic and weight goals out of the picture.

### 4.6 Limitations

Our findings may not be generalizable to all athletes as we had a relatively homogenous sample in terms of socio-economic status, geography, and ethnicity. However, our sample cuts across different grades, sports, and genders. This suggests that our work may be transferable to athletes in similar socioeconomic conditions and school systems. The number of non-binary adolescents in our sample was also small and not sufficient to fully understand this groups’ experiences. Despite this, our study is one of the first to consider the experiences of non-binary adolescents. Our analysis further did not explore the impact of culture or social influences on adolescents’ dietary habits, perceptions of sport or gender norms. As such, we can not speculate on who or what ultiamtely contributed to adolescents’ perceptions of their identities.

## 5 Conclusion

By exploring intersections between sport, gender, and dietary identities, we have obtained a deeper understanding of adolescent sport experiences. From the perspective of adolescent athlete and non-athletes, athletes recognize being muscular and eating protein, traditionally masculine physical and behavioral traits, as simply part of being an athlete. Despite this positive shift in view of eating and idealized bodies, adolescents still embodied a complicated relationship between their dietary and gender identities that might disadvantage different youth. As such, there is more work to be done to ensure that all youth in sport are equitably supported to develop dietary identities that focus on sport needs instead of gender norms.

## Data Availability

The raw data supporting the conclusions of this article will be made available by the authors, without undue reservation.

## References

[1] Merkel D. Youth sport: positive and negative impact on young athletes. Open Access J Sport Med. (2013) 4:151–60. doi: 10.2147/OAJSM.S33556, PMID: 24379720 PMC3871410

[2] Kristensen JÅ Haugen T Ommundsen Y. Supplement usage and doping attitudes in elite youth sports: the mediating role of dietary supplement acceptance. PLoS One. (2024) 19:e0297078–92. doi: 10.1371/journal.pone.0297078, PMID: 38300939 PMC10833512

[3] Kontele I Vassilakou T. Nutritional risks among adolescent athletes with disordered eating. Children. (2021) 8:10–2. doi: 10.3390/children8080715 PMC8394476 34438606

[4] Vani MF Pila E DeJonge M Solomon-Krakus S Sabiston CM. ‘Can you move your fat ass off the baseline?’ Exploring the sport experiences of adolescent girls with body image concerns. Qual Res Sport Exerc Heal. (2020) 13:671–89. doi: 10.1080/2159676X.2020.1771409

[5] Carl RL Johnson MD Martin TJ. Council on sports medicine and fitness. Promotion of healthy weight- control practices in Young athletes. Am Acad Pediatr. (2017) 140:1–13. doi: 10.1542/peds.2017-1871 28827381

[6] Sobal J Bisogni CA. Constructing food choice decisions. Ann Behav Med. (2009) 38:37–46. doi: 10.1007/s12160-009-9124-5 19787306

[7] Jovanov P Đorđić V Obradović B Barak O Pezo L Marić A. Prevalence, knowledge and attitudes towards using sports supplements among young athletes. J Int Soc Sports Nutr. (2019) 16:27–36. doi: 10.1186/s12970-019-0294-7, PMID: 31272457 PMC6611041

[8] Close GL Kasper AM Walsh NP Maughan RJ. “Food first but not always food only”: recommendations for using dietary supplements in sport. Int J Sport Nutr Exerc Metab. (2022) 32:371–86. doi: 10.1123/ijsnem.2021-0335 35279015

[9] Stokes EG Hughes R Shaw DM O’connor HT Beck KL. Perceptions and determinants of eating for health and performance in high-level male adolescent rugby union players. Sports. (2018) 6:49–58. doi: 10.3390/sports6020049, PMID: 29910353 PMC6027549

[10] Carson TL Tournat T Sonneville K Zernicke RF Karvonen-Gutierrez C. Cultural and environmental associations with body image, diet and well-being in NCAA di female distance runners: a qualitative analysis. Br J Sports Med. (2021) 55:433–7. doi: 10.1136/bjsports-2020-102559, PMID: 33139255

[11] Sabiston CM Doré I Lucibello KM Pila E Brunet J Thibault V. Body image self-conscious emotions get worse throughout adolescence and relate to physical activity behavior in girls and boys. Soc Sci Med. (2022) 315:115543–52. doi: 10.1016/j.socscimed.2022.115543, PMID: 36413860

[12] Bratland-Sanada S Sundgot-Borgen J. Eating disorders in athletes: overview of prevalence, risk factors and recommendations for prevention and treatment. Eur J Sport Sci. (2013) 13:499–508. doi: 10.1080/17461391.2012.740504, PMID: 24050467

[13] Messner MA. Gender ideologies, youth sports, and the production of soft essentialism. Sociol Sport J. (2011) 28:151–70. doi: 10.1123/ssj.28.2.151

[14] Hargreaves J. Sporting females: critical issues in the history and sociology of Women's sports In: Contemporary Sociology, ed. Chris Rojek vol. 24. London: Routledge (1994). 697–344.

[15] Adelphi University. Male vs female professional sports salary comparison. Sport Manag. (2023):1.

[16] Hargreaves J Anderson E. Gender and sexuality in the mediation of sport In: Handbook of sport, gender and sexuality. eds. Hargreaves J. Anderson E.. London: Routledge (2014). 1–54.

[17] Walton CC Rice S Gao CX Butterworth M Clements M Purcell R. Gender differences in mental health symptoms and risk factors in australian elite athletes. BMJ Open Sport Exerc Med. (2021) 7:e000984–90. doi: 10.1136/bmjsem-2020-000984, PMID: 33754081 PMC7939008

[18] Correia M Rosado A. Anxiety in athletes: gender and type of sport differences. Int J Psychol Res. (2019) 12:9–17. doi: 10.21500/20112084.3552, PMID: 32612783 PMC7110169

[19] Canadian Centre for Ethics in Sports. Power of sport: The true sport report 2022. Ontario, Canada: Guelph (2022).

[20] Koulanova A Sabiston CM Pila E Brunet J Sylvester B Sandmeyer-Graves A. Ideas for action: exploring strategies to address body image concerns for adolescent girls involved in sport. Psychol Sport Exerc. (2021) 56:102017–28. doi: 10.1016/j.psychsport.2021.102017

[21] Gumprich M Hare N. ANKORS Trans Connect. The Canadian non-binary youth in sport report. Vancouver, BC: (2023).

[22] Mancine RP Gusfa DW Moshrefi A Kennedy SF. Prevalence of disordered eating in athletes categorized by emphasis on leanness and activity type - a systematic review. J Eat Disord. (2020) 8:47–56. doi: 10.1186/s40337-020-00323-2, PMID: 33005418 PMC7523350

[23] Crenshaw K. Mapping the margins: intersectionality, identity politics, and violence against women of color. Stanford Law Rev. (1991) 43:1241–99. doi: 10.2307/1229039

[24] Wiens K Erdman KA Stadnyk M Parnell JA. Dietary supplement usage, motivation, and education in young Canadian athletes. Int J Sport Nutr Exerc Metab. (2014) 24:613–22. doi: 10.1123/ijsnem.2013-0087, PMID: 24667342

[25] Ricciardelli LA McCabe MP. A biopsychosocial model of disordered eating and the pursuit of muscularity in adolescent boys. Psychol Bull. (2004) 130:179–205. doi: 10.1037/0033-2909.130.2.179, PMID: 14979769

[26] Vartanian LR Herman CP Polivy J. Consumption stereotypes and impression management: how you are what you eat. Appetite. (2007) 48:265–77. doi: 10.1016/j.appet.2006.10.008, PMID: 17157957

[27] Schaillée H Derom I Solenes O Straume S Burgess B Jones V. Gender inequality in sport: perceptions and experiences of generation Z. Sport Educ Soc. (2021) 26:1011–25. doi: 10.1080/13573322.2021.1932454

[28] Burr V. Social Constructionism. 2nd editio. The Bloomsbury handbook of literary and cultural theory. London: Routledge; (2003). 1–240 p.

[29] Smith JA. Reflecting on the development of interpretative phenomenological analysis and its contribution to qualitative research in psychology. Qual Res Psychol. (2004) 1:39–54. doi: 10.1191/1478088704qp004oa

[30] Smith JA Osborn M. Interpretative phenomenological analysis In: Smit JA, editor. qualitative psychology: a practical guide to research methods. London: Sage Publications, Inc (2003). 51–80.

[31] Husserl E In: Nijhoff M, editor. Ideas pertaining to a pure phenomenology and to a phenomenological philosophy. First book. London: The Hague and New York (1983)

[32] Martínková I Parry J. An introduction to the phenomenological study of sport. Sport Ethics Philos. (2011) 5:185–201. doi: 10.1080/17511321.2011.602571

[33] Smith JA. Evaluating the contribution of interpretative phenomenological analysis. Health Psychol Rev. (2011) 5:9–27. doi: 10.1080/17437199.2010.510659

[34] Statistics Canada. 2021 Census of Population [Internet]. Census Profile. (2021). Available at: https://www12.statcan.gc.ca/census-recensement/2021/dp-pd/prof/details/page.cfm?Lang=E&SearchText=Tsawwassen&DGUIDlist=2021S05101570&GENDERlist=1,2,3&STATISTIClist=1&HEADERlist=0

[35] O’Halloran L Littlewood M Richardson D Tod D Nesti M. Doing descriptive phenomenological data collection in sport psychology research. Sport Soc. (2018) 21:302–13. doi: 10.1080/17430437.2016.1159199

[36] Michie S Marques MM Norris E. Theories and interventions in health behaviour change. Handb Heal Psychol. (2019):69–88. doi: 10.4324/9781315167534-6

[37] Slater J Falkenberg T Rutherford J Colatruglio S. Food literacy competencies: a conceptual framework for youth transitioning to adulthood. Int J Consum Stud. (2018) 42:547–56. doi: 10.1111/ijcs.12471

[38] Bem SL. The measurement of psychological androgyny. J Consult Clin Psychol. (1974) 42:155–62. doi: 10.1037/h0036215 4823550

[39] Callary B Rathwell S Young B. Insights on the process of using interpretive phenomenological analysis in a sport coaching research project. Qual Rep. (2015) 20:63–75. doi: 10.46743/2160-3715/2015.2096

[40] Modlinska K Adamczyk D Maison D Pisula W. Gender differences in attitudes to vegans/vegetarians and their food preferences, and their implications for promoting sustainable dietary patterns-a systematic review. Sustain For. (2020) 12:1–17. doi: 10.3390/su12166292

[41] Calzo JP Sonneville KR Haines J Blood EA Field AE Austin SB. The development of associations among body mass index, body dissatisfaction, and weight and shape concern in adolescent boys and girls. J Adolesc Health. (2012) 51:517–23. doi: 10.1016/j.jadohealth.2012.02.021, PMID: 23084175 PMC3479441

[42] Bennett EV Scarlett L Hurd Clarke L Crocker PRE. Negotiating (athletic) femininity: the body and identity in elite female basketball players. Qual Res Sport Exerc Heal. (2017) 9:233–46. doi: 10.1080/2159676X.2016.1246470

[43] Fostervold Mathisen TF Sundgot-Borgen C Anstensrud B Sundgot-Borgen J. Mental health, eating behaviour and injuries in professional dance students. Res Danc Educ. (2022) 23:108–25. doi: 10.1080/14647893.2021.1993171

[44] McKay Parks PS Read MH. Adolescent male athletes: body image, diet, and exercise. Adolescence. (1997) 32:592–602. 9360733

[45] Nelsen M. Which mixed team events will take place at Paris 2024? Olympic Games. (2024):1.

[46] Pelly FE Thurecht RL Slater G. Determinants of food choice in athletes: a systematic scoping review. Sport Med - Open. (2022) 8:99. doi: 10.1186/s40798-022-00461-8 PMC9188630 35689741

[47] Martinsen M Sundgot-Borgen J. Higher prevalence of eating disorders among adolescent elite athletes than controls. Med Sci Sports Exerc. (2013) 45:1188–97. doi: 10.1249/MSS.0b013e318281a939 23274604

[48] Sobal J Milgrim M. Gendertyping sports: social representations of masculine, feminine, and neither-gendered sports among US university students. J Gend Stud. (2019) 28:29–44. doi: 10.1080/09589236.2017.1386094

[49] Chapa DAN Hagan KE Forbush KT Perko VL Sorokina DA Alasmar AY. The athletes’ relationships with training scale (ART): a self-report measure of unhealthy training behaviors associated with eating disorders. Int J Eat Disord. (2018) 51:1080–9. doi: 10.1002/eat.22960, PMID: 30312490 PMC6519369

