# Supplementary material for: Exploring adolescents’ experiences navigating the intersection of their gender, sport, and dietary identities: an interpretative phenomenological study
Source: Front Nutr. 2024 Dec 19;11:1524135. doi: 10.3389/fnut.2024.1524135 (PMC11693689; doi:10.3389/fnut.2024.1524135)
Supplement: Supplementary file 1 [file Table_1.docx]

**SUPPLEMENTARY INFORMATION**

Table 1. Consolidated criteria for reporting qualitative studies (COREQ) 32-item checklist.

| **No.** | **Item** | **Description** | **Page #** |
| --- | --- | --- | --- |
| **Domain 1: Research team and reflexivity** | | | |
| Personal characteristics | | | |
| *1.* | Interviewer/ facilitator | Which author/s conducted the interview or  focus group? | 3 |
| *2.* | Credentials | What were the researcher's credentials? *E.g. PhD, MD* | 1 |
| *3.* | Occupation | What was their occupation at the time of the study? | 3 |
| *4.* | Gender | Was the researcher male or female? | 3 |
| *5.* | Experience and  training | What experience or training did the researcher have? | 3 |
| Relationship with participants | | | |
| *6.* | Relationship  established | Was a relationship established prior to study commencement? | 3 |
| *7.* | Participant knowledge of the interviewer | What did the participants know about the researcher? *E.g. Personal goals, reasons for doing the research* | 3 |
| *8.* | Interviewer characteristics | What characteristics were reported about the interviewer/facilitator? *E.g. Bias, assumptions,*  *reasons and interests in the research topic* | 3 |
| **Domain 2: Study design** | | | |
| Theoretical framework | | | |
| *9.* | Methodological orientation and theory | What methodological orientation was stated to underpin the study? *E.g. grounded theory, discourse analysis, ethnography, phenomenology, content analysis* | 3 |
| Participant selection | | | |
| *10.* | Sampling | How were participants selected? *E.g. purposive,*  *convenience, consecutive, snowball* | 3 |
| *11.* | Method of approach | How were participants approached? *E.g. face-*  *to-face, telephone, mail, email* | 3 |
| *12.* | Sample size | How many participants were in the study? | 3, Table 1 |
| *13.* | Non-participation | How many people refused to participate or  dropped out? What were the reasons for this? | Missing |
| Setting | | | |
| *14.* | Setting of data  collection | Where was the data collected? *E.g. home, clinic,*  *workplace* | 3 |
| *15.* | Presence of non-  participants | Was anyone else present besides the  participants and researchers? | 3 |

Developed from: Allison Tong, Peter Sainsbury, Jonathan Craig, Consolidated criteria for reporting qualitative research (COREQ): a 32-item checklist for interviews and focus groups, International Journal for Quality in Health Care. 2007;19(6): 349–357. https://doi.org/10.1093/intqhc/mzm042.

Table 2. EATing in a GENdered world (EatGen) Study interview guide and key probes.

| Topic | Questions asked |
| --- | --- |
| Dietary habits | 1. What are your favourite foods? |
|  | 2. What food(s) do you eat for protein? |
|  | 3. Do you ever alter what you eat to promote changes in your body? |
|  | 4. What is the most important factor to you when it comes to making/selecting foods? |
| Gender norms | 5. Can you define what the word masculine/feminine means to you? |
|  | 6. What would an example of a masculine/feminine food be? |
|  | 7. If you were to put masculine and feminine on a scale, where would you place yourself? |
| Food literacy | 8. How would you define a healthy/unhealthy food? |
|  | 9. Can you describe what the healthiest type of meal pattern might be? |
|  | 10. What does the term mindful eating mean to you? |
|  | 11. What does the term food literacy mean to you? |
|  | 12. Do you think it’s important to learn how to prepare food/how food impacts you? |
|  | 13. Where have you learned about how to prepare food/what food does for your body? |
|  | 14. What are you happy you learned /would you like to still learn about food? |
|  | 15. How could your experiences learning about food be improved? |
| Social impacts | 16. Do you feel that your friends play a role in influencing what you eat?  *Comfort level eating with friends, eating out, and eating with different friend groups* |
|  | 17. Do your parents play a role in what you eat?  *Level of autonomy, food expectations, and modeling of food tasks* |
|  | 18. Do you think the media influences what you eat?  *Food ads, food magazines/cookbooks, cooking shows, and food bloggers* |
|  | 19. Do your teachers play a role in influencing what you eat?  *Which class(es)* |
|  | 20. Of all the social influences, which do you think has the biggest role in what you eat? |
| Sport | 21. Growing up have you ever received advice on what you should eat for sports?  *What was it, who from, did you follow it* |
|  | 22. Have you ever felt pressure about the way you look in a uniform? |
|  | 23. What messages have you received from coaches/teammates surrounding body shape? |
|  | 24. Do you think food is important for sports? |
|  | 25. Do you ever alter what you eat for practices or games? |
|  | 26. What is one thing you’re happy you learned about nutrition and sport/still like to learn? |

Key probes for social influences (i.e., peers, parents, teachers, social media, coaches, and teammates) are *italicized*. All social influences were additionally probed for any changes after puberty (i.e., “changes in your body”) started and trust in nutrition advice given. Probes asking about opportunities to cook with parents and peers were also asked.
